# What Is a Clinical Lecture?

**Published:** 1915-06-05

**Authors:** 


### WHAT IS A CLINICAL LECTURE?

If we may judge from current medical publications the clinical lecture is one of the favourite forms in which authors delight to present and enshrine their teaching. Yet what authors understand by the term it is often very difficult to know. The difficulty is all the greater seeing that in addition to the literary categories above mentioned there is another—namely, the "lecture" pure and simple. Between this and the clinical lecture there must be, therefore, in the minds of those who employ the titles some appreciable and essential difference. Truth to tell, the difference is often hard to discover, at least to the uninitiated. Presumably the qualification "clinical" implies either something which is performed at the bedside or something which is observed from this standpoint. Now, the bedside is hardly a suitable spot for the delivery of a lecture. Hence a lecture designated as "clinical," if this qualification is to have any value at all, must mean teaching founded upon bedside observation and presented as an ordered and reasoned discussion of the facts which have been noted in the course of this study. It must therefore find its text in the sick man; in other words, it must have an individual character and must be an exposition of an actual and personal situation. Clearly, to attain its full value, the lecture must be addressed to an audience the members of which equally with the teacher have observed the clinical facts; or failing this, these must be effectively presented to the hearers. When these conditions are satisfied the qualification "clinical" is fully warranted, and such a lecture may be of a very high educational value. It deals with an individual diagnostic problem, seeks to expose the meaning of existing facts, and recognises the qualifications and difficulties which are attached to particular interpretations. In a word, it is concerned with exactly the kind of study which it is the lot of the practitioner daily to undertake.

There seems, however, very good reason to doubt whether the definition of the term "clinical lecture" as here presented is at all widely accepted. Authors offer to us the title, but under its cover they arrange very different material from that which the title suggests. This, indeed, is where the puzzle comes in, for very often, indeed, there would seem to be no line of demarcation between a lecture which is dignified as "clinical" and another which is not endowed with this qualification. A discourse on, say, jaundice or on hemiplegia, with a complete examination of varieties, causes, symptoms, differential diagnosis, pathology, and treatment we can understand, but why should the performance be called a "clinical" lecture? Sometimes this form of exposition does actually take place at the bedside, inasmuch as the sick man is brought into the lecture room for the purpose. But his presence there has little relation to the main purpose of the discourse, which is not the individual situation of the patient, but a systematic exposition of a selected area of medical knowledge. It may be magnificent; but a clinical lecture in any reasonable sense of the term it certainly is not. We have even heard of a professor who occupied the session with what he called "clinical lectures" on the properties, preparations, physiological and pathological actions, and therapeutic uses of arsenic; but we should say that unless he gave the medicine freely to his patients and demonstrated the effects to the students as he went along his title was wanting in justification. To prepare a series of discourses in advance and then to label them "clinical" because they are delivered in a hospital or by a member of a hospital staff is an abuse of language. It is, we hold, in the interests of accuracy that the "systematic" lecture and the "clinical" lecture should be recognised as different both in the kind of text from which they start and in the ambition which they seek to compass. Each has its place and purpose, but the one ought not to be confounded with the other.

